# Understanding Sensory Nerve Mechanotransduction through Localized Elastomeric Matrix Control

**DOI:** 10.1371/journal.pone.0004293

**Published:** 2009-01-28

**Authors:** Yi-Wen Lin, Chao-Min Cheng, Philip R. LeDuc, Chih-Cheng Chen

**Affiliations:** 1 Institute of Biomedical Sciences, Academia Sinica, Taipei, Taiwan; 2 Department of Mechanical and Biomedical Engineering, and Biological Sciences, Carnegie Mellon University, Pittsburgh, Pennsylvania, United States of America; Flinders University, Australia

## Abstract

**Background:**

While neural systems are known to respond to chemical and electrical stimulation, the effect of mechanics on these highly sensitive cells is still not well understood. The ability to examine the effects of mechanics on these cells is limited by existing approaches, although their overall response is intimately tied to cell-matrix interactions. Here, we offer a novel method, which we used to investigate stretch-activated mechanotransduction on nerve terminals of sensory neurons through an elastomeric interface.

**Methodology/Principal Findings:**

To apply mechanical force on neurites, we cultured dorsal root ganglion neurons on an elastic substrate, polydimethylsiloxane (PDMS), coated with extracellular matrices (ECM). We then implemented a controlled indentation scheme using a glass pipette to mechanically stimulate individual neurites that were adjacent to the pipette. We used whole-cell patch clamping to record the stretch-activated action potentials on the soma of the single neurites to determine the mechanotransduction-based response. When we imposed specific mechanical force through the ECM, we noted a significant neuronal action potential response. Furthermore, because the mechanotransduction cascade is known to be directly affected by the cytoskeleton, we investigated the cell structure and its effects. When we disrupted microtubules and actin filaments with nocodozale or cytochalasin-D, respectively, the mechanically induced action potential was abrogated. In contrast, when using blockers of channels such as TRP, ASIC, and stretch-activated channels while mechanically stimulating the cells, we observed almost no change in action potential signalling when compared with mechanical activation of unmodified cells.

**Conclusions/Significance:**

These results suggest that sensory nerve terminals have a specific mechanosensitive response that is related to cell architecture.

## Introduction

Mechanical force is known to affect a diversity of physiological areas at the cellular level including cardiac, fibroblast, bone, and vascular cells [1]–[3]. Mechanotransduction is a topic of increasing research interest, particularly to those in the neural sciences, due to the ability of physically based forces to induce neuronal changes that are directly responsible for a host of complex and integrated responses. Mechanoreceptors of sensory neurons localize in specialized or encapsulated nerve terminals, providing a mechanism of response to pain, touch, pressure, vibration, vessel stretch, and propriocecption [4]. However, while initial evidence suggests that mechanobiology applies strictly to nerve terminals in neurons, the manner in which these terminals sense and respond to mechanical signals is still not well understood. Furthermore, previous studies investigating the molecular mechanisms of mechanotransduction in sensory neurons (by adopting neurite-free neurons to stimulate nerve terminals) used mechanical forces such as compression and hypo-osmotic stretch [5], [6]. This stimulus was applied directly on the soma of acute dissociated dorsal root ganglion (DRG) neurons and generated an inward current detected via patch clamp recording [5]–[10]. Two recent studies measured pressure-activated currents on nerve terminals through the use of a glass pipette or a pressure jet [11], [12]. As this is a nonspecific means of mechanically stimulating neural cells, this does not replicate a direct link to structural interactions, e.g., focal adhesions, found in mammalian cells [13]. To our knowledge, no research attempt has been reported that examined stretch-activated mechanotransduction on neurites in dissociated neurons through a specific adhesion mechanism while providing control over the cell-matrix mechanical properties.

In our study, we first focused on developing an *in vitro* method to probe stretch-activated mechanotransduction and cytoskeletal structural links for nerve terminals in neurons. This allowed us to investigate the coupled behavior of mechanical stimulation and substrate interactions, with respect to cell structure, for affecting the critical neural function of action potential (AP) firing. To provide control over the mechanical stimulation and the cell-matrix interactions, we used polydimethylsiloxane (PDMS), which has a highly cross-linked three-dimensional structure, and offers high elongation properties with a relatively low modulus. PDMS is composed of a silicone T-resin cross-linked by a mixture of vinyl-terminated PDMS (base) and trimethylsiloxy-terminated polymethylhydroxosiloxane polymers (curing agent) [14]. PDMS can be modified to have various elasticity properties, which is useful when using force as an influential parameter to understand cell signaling, since it allows for precise modulation of the PDMS down to a single kPa, an elasticity similar to that found in native tissue [15]. The ability to modulate elasticity in the PDMS also provides for control over the amount of deformation that the substrate, and cells attached to the substrate, might experience under controlled deformations. We have, through this simple approach, leveraged material characteristics to emulate physiologically relevant interactions in nerve terminals experiencing mechanical stimulation for probing mechanotransductive response in DRG neurons.

## Results

### Probing mechanotransduction in neurites through elastomeric matrix control

**Figure 1:**
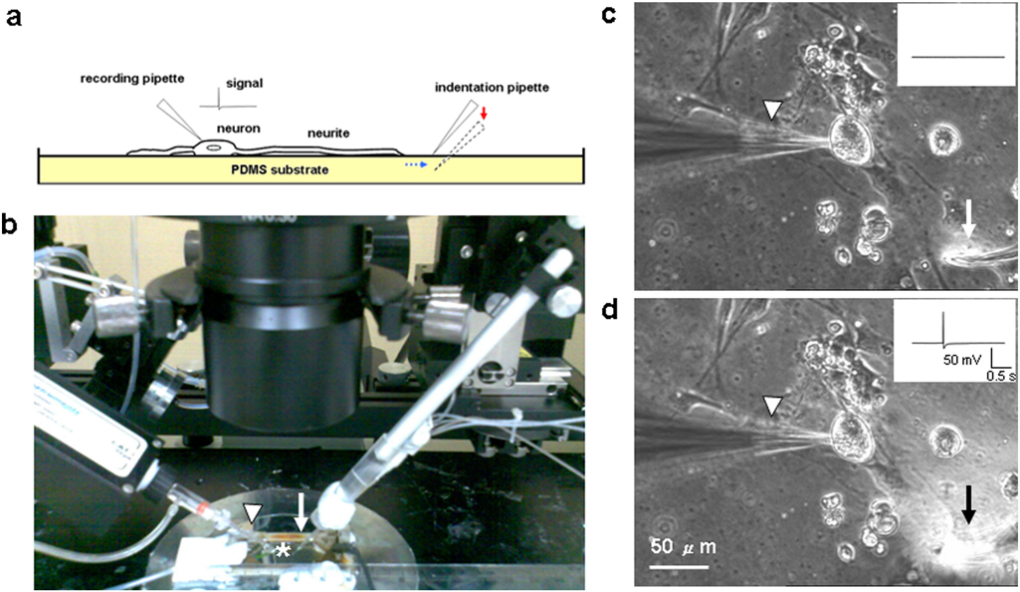
Experimental setup for using elastomeric substrates to probe mechanotransduction in neurites. a,Schematic of the mechanical stretching imposed on neurites in the recording chamber. DRG neurons cultured on a PDMS substrate were recorded with a recording pipette. Force on the neurites was generated by indenting the PDMS substrate with a glass pipette. The force from the indenting pipette was transmitted through the substrate to the cell. The signal from a DRG neuron was recorded through a whole-cell patch clamp set-up. b, An image of the system used to record stretch-evoked AP from neurites. The recording was performed in a recording chamber (asterisk) constantly perfused with ACSF. The neuron was connected to a recording pipette (white arrowhead) that was attached to a pre-amplifier. The neurites were stretched by the pipette (white arrow) indentation that was controlled with a micromanipulator. c, When the indentation pipette (white arrow) was placed on the surface of PDMS substrate near the neurite, no AP was generated (inset). d, As the pipette indented the PDMS through a vertical displacement (micromanipulator), a change in intensity in the differential interference contrast (DIC) image at the location of the pipette was observed (black arrow). This indentation, which imposed a force on the attached cell, evoked an AP (inset).

One goal was to build a PDMS culture-recording setup that allows for distal force application to nerve terminals through an elastic deformation with simultaneous recording of the consequent AP response on a neuron soma. To provide a system that would allow us to impose these mechanical forces, we first needed to successfully integrate our cell culture methodology with the indentation pipette force procedure and AP measuring techniques. First, PDMS substrates with low stiffness were fabricated to provide an elastic connection similar to what has been observed in living organisms [16]. Next, we coated the PDMS with either fibronectin or poly-L-lysine and cultured neurons on the modified elastic surface; neurite out-growths were observed using this approach. To impose mechanical force, we used a micromanipulator to bring a blunt pipette into contact with the PDMS and deform the PDMS through a vertical displacement of the pipette at a location that was near to, but not on, the neurite. Through this approach, we mechanically stimulated the neurite extension, but did not impose non-specific mechanical stimulation on the cells; a schematic of this method is shown in Figures 1a and 1b. While the displacement of the pipette was vertical, the deformation of the substrates caused a stretching of the cell along its cell-substrate interface, as the cell was attached to the substrate (Fig. S1). Mechanical stimulation on this single neurite increased with increases in the indentation depth (the relationship of indentation depth to force application is described in Fig. S2). Thus, we were able to apply force on DRG neurites via specific attachments due to the molecular coating-substrate interactions. This also minimized direct probe interaction with the cells, which could be detrimental to cell function. We next demonstrated that the condition of whole-cell patch recording was stable and reliable when the PDMS was indented. The effect of indentation on serial or input resistance was minimal (<30%). In addition, the indentation did not rupture the soma or neurites via visualization with lucifer yellow (n = 5, Fig. S3). Before and during the application of mechanical stimulation, we recorded the evoked AP or the change of membrane potential on the soma (Fig. 1c,d).

### Effect of ECM and neurites on stretch-activated mechanotransduction

**Figure 2:**
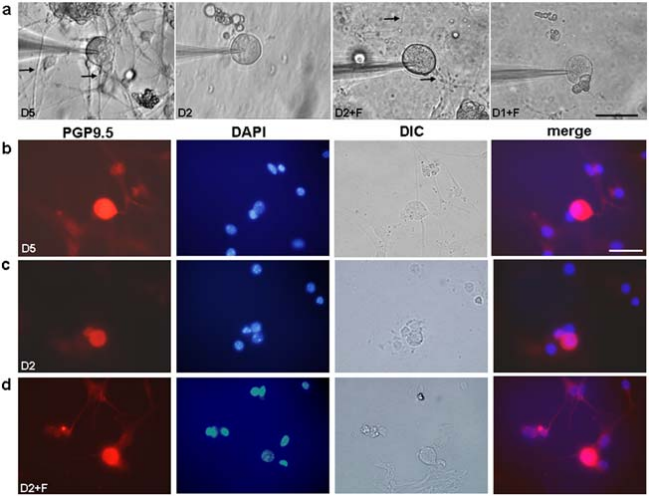
Attachment and growth of DRG neurons on PDMS substrates with defined ECMs. a, Patch recorded DRG neurons cultured on PDMS substrates coated with poly-L-lysine showed no neurite outgrowth in day-2 culture (D2), but extended neurites were observed in day-5 culture (D5). DRG neurons cultured on PDMS substrates coated with fibronectin showed no neurite outgrowth in day-1 culture (D1+F), yet displayed extended neurites in day-2 culture (D2+F). Arrows indicate the neurites. b–d, To distinguish neurons from glial cells, the cells were stained for protein gene product 95. (PGP 9.5), which is an ubiqutin C-terminal hydrolase specifically expressed in most neurons and known to be present in neurites of DRG [40]. b, DRG neurons cultured on poly-L-lysine-coated PDMS substrates for 5 days showed extended neurites and presence of PGP 9.5 (red pseudo-color) along with DIC and DAPI imaging, indicating the cell nucleus (blue pseudo-color). c, PGP 9.5-positive DRG neurons cultured on poly-L-lysine-coated PDMS substrates at day-2 showed no neurite outgrowth. d, PGP 9.5-positive DRG neurons cultured on fibronectin-coated PDMS substrates showed extended neurites at day-2. Scale bar, 50 µm.

**Figure 3:**
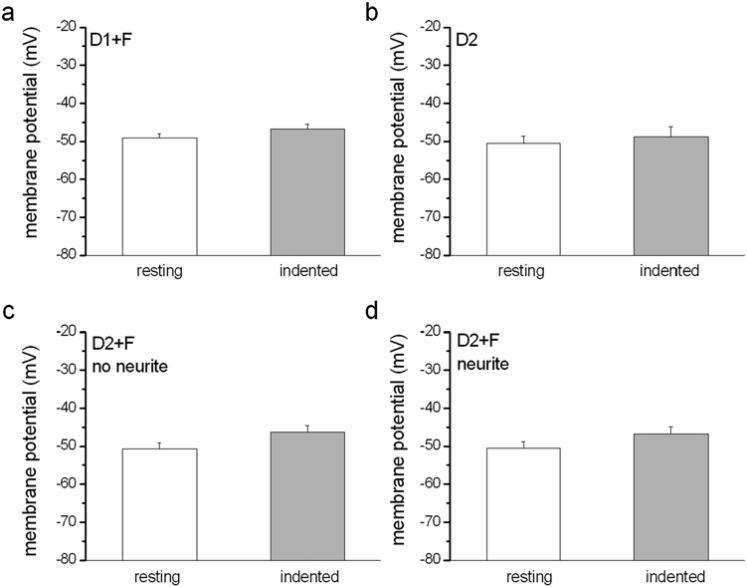
Membrane potentials of neurons without stretch-activated AP. In a current clamp mode, membrane potentials were recorded before and during mechanical stimulation with a 125 µm depth of indentation. a, Neurite-free DRG neurons in 1-day culture with fibronectin (n = 38, p>0.05). b, Neurite-free DRG neurons in 2-day culture with poly-L-lysine (n = 19, p>0.05). c, Neurite-free DRG neurons in 2-day culture with fibronectin (n = 12, p>0.05). d, Neurite-bearing DRG neurons in 2-day culture with fibronectin (n = 10, p>0.05).

**Figure 4:**
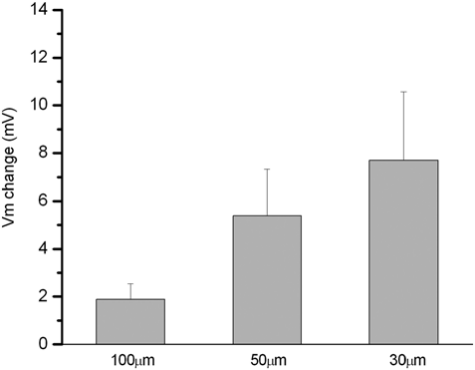
Effect of indentation distance from soma. Whole-cell patch recordings were performed in DRG neurons in 2-day culture with poly-L-lysine. All neurons were neurite-free. In the current clamp mode, the change of membrane potential was recorded when a maximal indentation depth (125 µm) was applied to the PDMS at a distance of 100 µm, 50 µm, or 30 µm away from the soma. When the indentation was placed on the soma directly, all neurons fired an AP (Fig. S5). p>0.05 comparison between any two distances, n = 5.

The response of cells is often directly related to ECM interactions occurring between the cell and the substrate [17]. Thus, we probed these interactions by using poly-L-lysine and fibronectin, which have different cell adhesion characteristics. We found that the specificity of the cell-to-ECM interactions had a pronounced effect on the response of neurites (Fig. 2). We quantified the AP for cells when cultured with different substrate conditions to probe neurite response (Table 1). Only the neurite-bearing neurons can display an evoked AP. In contrast, for neurite-free neurons, neither was an AP evoked nor was membrane potential altered during the application of mechanical stimulation at a 100 µm distance from the soma (Fig. 3). Even when the indentation was close to the soma (within 30 µm), the mechanical stimulation only caused limited changes of membrane potential (<10 mV) for neurite-free neurons (Fig. 4).

**Table 1:** Summary of stretch-activated APs evoked in DRG neurons with defined culture conditions.

Culture condition	D5	D2	D2+F	D1+F
Number of tested neurons	40	20	30	40
Cell size (µm)	29.3±1.7	30.2±1.4	31.5±0.8	32.1±0.9
Stretch induced AP (%)	14 (35%)	0	8 (26.7%)	3 (7.5%)
AP indentation depth (µm)	57.3±8.8**	NA	80.7±10.9	107.6±3.4**
Indentation force (µN)	113.7±17.4**	NA	160.3±21.7	213.6±6.8**
Membrane potential (mV)	−53.7±1.1	−52.9±1.7	−55.1±1.6	−54.1±1.2

The following result parameters were captured: D5, 5-day culture with poly-L-lysine; D2, 2-day culture with poly-L-lysine; D2+F, 2-day culture with fibronectin; and, D1+F, 1-day culture with fibronectin. Neuron diameters were determined, and the percentage of cells that responded to the indentation experiments with an AP signal was determined. AP indentation depth indicates the indentation depth needed to induce an AP for each condition. The indentation force at the neurite site is determined based on the equations provided in the Supplementary Information (Fig. S2). AP, action potential; NA, not applicable. **p<0.01 comparison with values of D2+F.

We found that mechanical stimulation of neurites cultured on PDMS substrates that were coated with poly-L-lysine induced an AP in only 35% of DRG neurons (n = 40) after day 5 of cell culture (Fig. 2a,b and Table 1). Indentation of the PDMS did not induce an AP in neurons without neurite outgrowth by day 2 (n = 20), although most neurons did not exhibit outgrowth of neurites at this time (Fig. 2c). In contrast, we found that fibronectin coating on PDMS substrates largely facilitated neurite outgrowth of cultured DRG neurons, with neurite extensions visible after only 2 days of culture (Fig. 2d). Fibronectin on the other hand did not promote an increase in the number of glia cells by day 2 (Table S1). Furthermore, at only 2 days of culture on fibronectin-coated PDMS, 26.7% of the DRG neurons (n = 30) had an AP under mechanical stretching, which was an improvement from 7.5% of the DRG neurons (n = 40) after 1 day, even with limited observable neurite outgrowth (Table 1). Not only did DRG neurons cultured on fibronectin extend neurites between days 1 and 2, but the number of them that responded to stretch via AP also increased. Furthermore, a lower threshold for the induction of an AP in these cultures was observed (213 vs. 160 µN). It is noted that although the stretch-induced AP was only found in neurons with neurite-outgrowth, a subset of neurite-bearing neurons (10/18 in D2+F group) did not display either AP induction or a change of membrane potential in response to stretch (Fig. 3d). Due to a robust AP response found in 44.4% (8/18) of neurite-bearing neurons, we subsequently used fibronectin coating after 2 days for additional studies.

### Involvement of cytoskeletal structure

**Figure 5:**
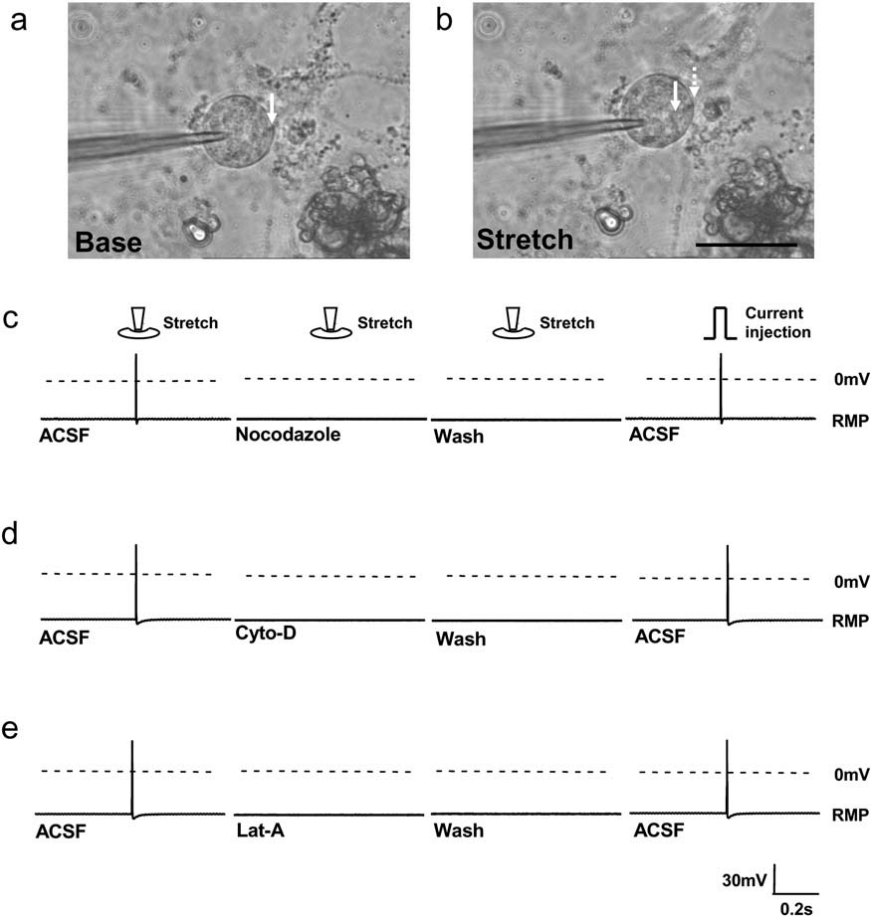
Examining stretch-activated mechanotransduction in neurites with respect to cytoskeletal structure. a–b, DIC images of neurons stretched in the direction of the indentation of the PDMS substrate. The edge of the neuron was marked with a solid arrow before the stretch and a dashed arrow after the stretch (the indentation pipette is not in the image). c, A baseline signal for each cell was recorded, and then an indentation was applied. Cytoskeletal modification agents were perfused into the system, and then a second indentation was applied. The cytoskeletal modifiers were then washed out using a continuous flow over 3 minutes followed by another indentation. A current injection (2 nA) was introduced afterwards while the AP was being recorded. Nocodazole (1 µg/ml), which disrupts microtubules, blocked stretch-activated AP but not the current injection-induced AP (n = 8). d, Cytochalasin-D, which disrupts actin filaments (1 µg/ml), blocked the stretch-activated AP but not the current injection-induced AP (n = 8). e, Latrunculin-A (1 µg/ml), which inhibits actin polymerization, blocked the stretch-activated AP but not the current injection-induced AP (n = 8). ACSF, artificial cerebrospinal fluid; RMP, resting membrane potential. Scale bar, 50 µm.

Since the cytoskeleton in many cell types is directly related to the ECM and mechanical response [1], [2], we next probed the effects of cytoskeletal structure in the mechanotransductive response of neural cells, using the approach outlined in Figure 5. We first investigated microtubules since they are a significant component of neurons and are heavily involved in many signaling pathways [18]. We mechanically stimulated neurons and recorded the AP in a manner similar to the previous experiments outlined in Figure 1. We then used nocodazole to interfere with the polymerization of microtubules [19] and recorded the AP signaling under mechanical stimulation. We observed that nocodazole abrogated the stretch-evoked action potentials in 100% of the tested neurons (Fig. 5c). As it was evident that at least one of the cytoskeletal components, i.e., microtubules, influenced AP firing, we then proceeded to examine another major cytoskeletal constituent, actin [19]. We first mechanically activated the neurons and then incubated them with cytochalasin-D or latrunculin-A, both of which affect the polymerization of actin filaments. The stretch-evoked action potentials were suppressed in all neurons following this actin cytoskeleton modification (Fig. 5d,e). To examine the reversibility of the signal, we followed the addition of the agent and mechanical stimulation with a continuous washing procedure (lasting for 3 minutes) to remove the cytoskeletal modifiers via bath-perfusion as previously published [20]. We sought to probe the response of the neuron after the wash-out to investigate whether they remained functional for AP signaling. After the washing procedure, we mechanically stimulated the cell again. This time, no AP firing was observed, although the cells were still responsive as determined by a follow-on current injection to the cell, which evoked a well characterized AP response. We further confirmed that the effect of nocodazole, cytochalasin-D, and latruculin-A were due to the depolymerization of the cytoskeleton and not the inhibition of voltage-dependent ion channels, as the neurons were still capable of firing APs through current injection even after being subjected to inhibiting cytoskeletal modifiers (Fig. S4). None of cytoskeleton modification agents altered the resting membrane potential of neurons.

### Effect of mechanosensitive channel blockers

**Figure 6:**
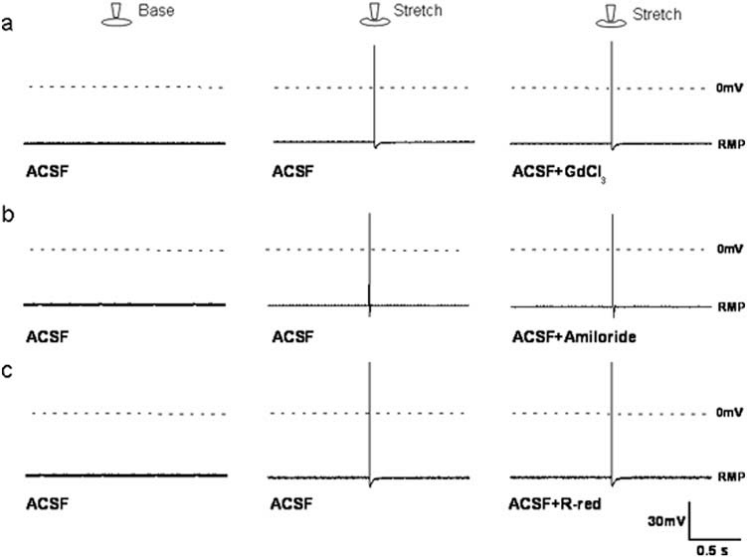
The response of stretch-activated mechanotransduction in neurites to mechanosensitive (MS) channel blockers. Common MS channel blockers were used to probe their effect on AP signaling. A baseline signal for each cell was first recorded with no stimulation provided. This was followed by recording the AP signal following the application of force from an indentation with no channel blocker being used. Next, the blocker was applied to the neurite, and another indentation was made; the AP was recorded during both of these events. (a) The stretch-activated AP was not blocked by GdCl_3_ (100 µM, n = 8), (b) amiloride (100 µM, n = 8), or (c) ruthenium red (5 µM, n = 8).

After determining that mechanical response was linked to the specificity of the ECM and the cytoskeleton, we were interested in examining whether there was a link involving mechanosensitive ion channels. To accomplish this, we used known mechanosensitive ion channel blockers (gadolinium chloride, amiloride, ruthenium red) that have been shown to inhibit most of the mechanosensitive current in neurite-free DRG neurons [5], [6]. Application of these blockers did not show any inhibitory effect on stretch-activated conductance of neurites cultured on fibronectin-coated PDMS substrate (Fig. 6). We found that gadolinium ions (Gd^3+^) did not block the stretch-evoked action potentials in DRG neurons (Fig. 6a). While gadolinium ions, a non-specific blocker of mechanosensitive ion channels in most cell types including neurons and non-neuronal cells [5], [6], [21]–[23], blocks mechanotransduction in most sensory neurons *in vitro* [5], [6], [11], many studies have shown that stretch-evoked afferent fiber responses are insensitive to gadolinium *in vivo* [24], [25]. For *in vivo* studies, the inhibitory effect of gadolinium on afferent mechanotransduction has only been demonstrated in specialized primary afferents such as those of the knee joint and the carotid baroreceptor nerve [26], [27]. In our recording system, gadolinium ions did not block the stretch-activated action potential in neurites (Fig. 6a). We opine that the mechanosensitive channels of neurites on elastic substrates coated with fibronectin may differ from those grown on more rigid glass or Petri dishes, or those using poly-L-lysine rather than fibronectin. In addition to investigating gadolinium, we examined amiloride and ruthenium red. Amiloride (or benzamil), does not block mechanically activated current in *in vitro* recording [6], but does inhibit mechanotransduction in airway afferents, intraganglionic laminar endings of vagal tension receptors, and knee joint afferents [24]–[26]. Our system did not reveal any amiloride-sensitive mechanotransduction (Fig. 6b); this may be related, again, to the substrate stiffness and ECM interactions *in vivo*. While rutheunium red and other TRP channel blockers inhibit mechanically activated current in soma [6] or slow-adapting current in neurites of isolated DRG [11], our results using ruthenium red did not reveal inhibition of mechanically induced signaling (Fig. 6c). While the neurites showed specific mechanosensitive responses when probing their AP, the blocking of mechanosensitive channels had little effect on the AP response.

## Discussion

Our studies have shown that neural action potential firing through nerve terminals is linked to specific mechanical deformation and extracellular matrix interactions. Since ECM-interfacing neurite outgrowth on soft substrates has been linked to integrins [28], [29], this suggests the potential for the transmission of mechanical stimulation through transmembrane integrins in nerve terminals. The integrin pathways is known to be critical in a diversity of mammalian cells types as being part of the focal adhesion complexes that are linked to mechanotransduction [30], [31]. While the exact mechanism for the activation is not known even in highly studied cells in mechanical environments such as endothelial and cardiac cells, the overall link is known to exist due to the signaling pathways such as MAPK that are activated under mechanical stimulation. The presence of direct molecular links to integrins such as with RGD in supporting physiological structures including the basement membrane is one reason for this mechanistic link to be pursued. As the integrins are transmembrane they provide a link from the extracellular interactions directly into the intercellular structures such as the cytoskeleton. The cytoskeleton has been shown to be linked to the responses in a diversity of cells and responses [1], [32], [33]. Thus, disrupting these structural links within living cells often alters or abrogates critical cell functions. This has similarities in AP response where while the exact mechanism has not been demonstrated, the response has been shown to be related to mechanical stimulation and also the cytoskeleton here. While the actin/microtubule inhibitors effectively abolished the stretch-induced AP response in all neurite-bearing neurons, a washout of the drugs did not confer a reversible AP response to stretch (Fig. 5). The reason is that although the drugs were washed out, the effects of the actin/microtubule inhibitors were not reversible in our recording system, which was usually completed in 30 minutes and not reliable beyond 1 hour since actin filaments and microtubules need extended time (hours) to repolymerize into more fully developed and functional forms.

While previous studies on mechanosensory transduction used sensory neurons cultured on coverslips coated with non-specific attachment proteins including poly-L-lysine, such conditions for other cell types have shown significantly different responses [34]. In each of our studies, we found that neurons cultured on PDMS coated with fibronectin promoted neurite extensions (Fig. 2). The neurons in these cultures also exhibited a lower threshold of stretch-activated action potentials when compared to neurons cultured upon a poly-L-lysine coating, implying that cell-ECM interactions are extremely important in neural mechanotransduction.

While displacements on nerve terminals with poly-L-lysine/laminin-coated coverslips have previously been observed to display mechano-sensitive currents 92% of the time [11], only 44.4% (8/18 of D2+F) of neurite-bearing neurons cultured on fibronectin-coated PDMS fired AP via distal stretch. The previous study used changes in pressure as the mechanical stimulus and there was not direct contact/linkage to the cell. Thus this approach had very little specificity in terms of ECM interactions. In addition, the discrepancy between these responses and the previously published experiments [11] could be due to the intensity of mechanical stimulation. The pressure that was used in the previous stimulation scenario was likely greater than the applied stress levels used in these studies, which may, at least partly, have been due to the stimulation mode(s) chosen (pressure vs. stretch). In addition, the substrate stiffness (glass vs. soft PDMS) could have had an influence, as stiffness has been shown to affect a variety of cell responses from motility to differentiation [35], [36]. Furthermore, while mechanotransduction *in vivo* is functionally executed at the sensory nerve terminals projecting to peripheral tissues and cell bodies of primary sensory neurons have not been shown to be ordinarily mechanosensitive, most of the previous studies used neurite-free DRG neurons to probe the mechanical activation in testing whole-cell current response *in vitro* [5]–[10]. One advantageous manifestation of our culture system is that it implements an elastomeric substrate coated with fibronectin, which likely is more accurate in mimicking the *in vivo* environment for nerve terminals to sense mechanical forces.

In the PDMS culture-recording setup, although D5 groups had the highest response rate for mechanical stimulation when compared with the other groups, the culture condition also show a high density of neurites and glia cells (Fig. 2). Therefore, we using this created a challenge for stretching a single neurite and avoiding the glia cells. In contrast, the neurons of the D2+F group provided two major advantages to probe the distal force-mediated mechanosensory transduction. First, fibronectin promoted neurite outgrowth on PDMS by day 2 and the neurite outgrowth was in a low-density and trackable level (Fig. 2). We could easily stretch a single neurite through a non-contact indentation. Also, the indentation was applied 10–15 µm next to a neurite, so that the neurite would receive similar mechanical forces when the same indentation depth was applied. Second, fibronectin did not promote proliferation of glia cells on PDMS by day 2, since the ratios of glia cells to neurons in D2 and D2+F groups were similar, 2.7 vs. 2.8 respectively (Fig. 2 & Table S1). These densities of glia cells allowed us to avoid direct indentation on glia cells and minimize the effect of glia cells on the mechanosensory transduction. Moreover, the mechanical stimulation had little effect on membrane potential change since the indentation was displaced 100 µm away from the soma, in which the distal mechanical force could only alter less than 3 mV of membrane potential on a neurite-free neuron, even the neuron was surrounding with glia cells (Fig. 3 & Fig. 4). When the neurite-free neurons received larger mechanical force as the indentation was closer, they may have a larger change in membrane potential. Nevertheless, the neurite-free neurons only altered less than 10 mV of membrane potential when the indentation was displaced in 30 µm distance (Fig. 4). Likewise, the effect of distal force on glia cells should be minimal. Although glia cells and neurons were side-by-side on PDMS substrate (Fig. 2), when the indentation was displaced adjacent to glia cells, no AP was induced in neurite-free neurons in D2, D1+F, and D2+F groups. However, although unlikely, we still cannot totally exclude a possibility that the mechanotransduction of neurites was an effect of gliatransmitters, which can be released when the glia cells receive enough mechanical stimulation [37].

We conclude that the distal force-mediated mechanosensory transduction we observed in neurites is fibronectin-influenced, and that the cytoskeleton is both involved and required for AP firing. These findings have implications in a diversity of fields including mechanotransduction in neurons, neuron-material interactions, and neural tissue engineering.

## Materials and Methods

### PDMS

Polydimethylsiloxane (PDMS, Sylgard 184) was purchased from Dow Corning Corp (Midland, MI, USA) and prepared with a 35∶1 ratio of base to curing agent. The elastic modulus of PDMS with this ratio has a Young's modulus of approximately 88 kPa [16].

### DRG primary culture

CD1 mice (8 to 12 weeks old) were used for DRG primary culture. The usage of these animals was approved by the Institute Animal Care and Use Committee of Academia Sinica and followed the Guide for the use of Laboratory Animals (National Academy Press). Mice were euthanized by the use of CO_2_ to minimize suffering. Total DRG were acutely dissociated and processed as described [38]. The cells were seeded on a PDMS layer on the top of a coverslip and coated with poly-L-lysine (0.1%; Sigma, St. Louis, MO) or fibronectin (10 µg in 1 ml PBS; BD Biosciences, San Jose, CA), then cultured in a Petri dish using Dulbecco's modified Eagle's medium containing 1% Penicillin/Streptomycin, and 10% fetal calf serum. Cell cultures were maintained in a 5% CO_2_ incubator at 37°C for 1 to 5 days.

### Electrophysiology

Whole-cell patch recordings of DRG neurons were performed as described previously [38]. The patch pipettes (64-0792, Warner Instruments, Hamden, CT) were prepared in 1–5 MΩ and filled with internal solution containing 100 mM KCl, 2 mM Na_2_-ATP, 0.3 mM Na_3_-GTP, 10 mM EGTA, 5 mM MgCl_2_, and 40 mM HEPES, adjusted to pH 7.4 with KOH. As the external solution, we used artificial cerebrospinal fluid (ACSF), which contained 130 mM NaCl, 5 mM KCl, 1 mM MgCl_2_, 2 mM CaCl_2_, 10 mM glucose, and 20 mM HEPES, adjusted to pH 7.4 with NaOH. A current clamp mode was used to record action potentials evoked by mechanical forces. The bridge was balanced for current clamping recording and the data were discarded if the serial resistance or input resistance varied >30% from the base recording (non-stretched state) during the indentation [39]. Chemicals were purchased from Sigma (St. Louis, MO).

### Mechanical stimulation

We applied a mechanical force on the PDMS after first confirming that the neuron could be excited through the introduction of a voltage change and measurement of an AP. A flamed polished pipette (tip diameter ∼4 µm) was used to indent the PDMS for the generation of mechanical stimulation through deformation of the substrate. The indentation pipette was located approximately 100 µm away from the main cell body of the recorded neuron and imposed a displacement on the PDMS where there were no glia cells. If a visible neurite was observed, the indentation was displaced adjacent to (∼10–15 µm) the neurite extension, which allowed us to avoid nonspecific contact with the neurite and also provided specific control over the amount of indentation that was imposed on the PDMS for stimulating the cell. Indentation was controlled through the use of a micromanipulator (EMM-3SV, Narishige, Tokyo, Japan) positioned at an angle of 45 degrees to the PDMS surface. The displacement was applied with a 10.42 µm step until an AP response occurred or a maximum total displacement of 125 µm was reached. When an indented depth activated an AP response, we always applied the same indentation force again to determine whether the stretch-activated AP was repeatable. The duration of a displacement lasted less than 1 second. A minimum of 30 seconds was used between each indentation [7].

### Chemical modulators for channels and the cytoskeleton

Gadolinium chloride (100 µM), amiloride (100 µM), and ruthenium red (5 µM) were prepared in ACSF and bath-perfused while the indentation was applied. Nocodazole (1 µg/ml), cytochalasin-D (1 µg/ml), and latrunculin-A (1 µg/ml) were prepared in ACSF and bath-perfused to the recording chamber. The cytoskeleton modification agents were incubated with the cultured neurons for 10 minutes before the indentation was applied [20]. After the drugs were applied, the recording chamber was washed with ACSF for 3 minutes and then a 2-nA square pulse was delivered to evoke an action potential.

### Microscopy and immunofluorescent staining

The differential interference contrast images of the recorded neurons were captured with a Charge-Coupled Device camera (XC-ST50, Sony, Japan) on an inverted microscope (IX71, Olympus, Tokyo, Japan). The fluorescent images were captured with a digital camera (Diagnostic Instruments, MI) on an Axiovert inverted fluorescent microscope (Axiovert 200, Zeiss, Germany). Cultured DRG neurons were fixed with 4% paraformaldehyde, and then incubated with PBS containing 10% bovine serum albumin for blocking and a primary antibody for protein gene product 9.5 (Chemicon, Temecula, CA) at 4°C overnight. The secondary antibody used was 6 µM Alexa Flour® 594 rabbit anti-guinea pig IgG (Invitrogen, Carlsbad, CA), which was applied for 2 hours. The cells were mounted with VECTASHIELD-DAPI (Vector, Burlingame, CA) and imaged using a 63× high numerical aperture oil immersion objective to examine morphology and fluorescent marker distribution.

### Statistical Analysis

Results are presented as the mean±SEM. One-way ANOVA tests were applied for independent sample comparison [38]. A non-parametric Mann-Whitney method was applied for independent sample comparison in Figure 4. A p<0.05 was considered significant.

## Supporting Information

Figure S1 Stretch neurites on PDMS substrate (movie). A neurite-bearing DRG neuron in day-2 culture with fibronectin was stretched through indentation on the PDMS. The duration of the indentation lasted for 0.64 seconds. The movie is composed of 16 frames.
(3.87 MB SWF)

Figure S2 Calculation of indentation forces. We generated a stretching force on a single neurite through indentation with a glass pipette into the soft PDMS surface. The indentation depth, h, was determined using a micromanipulator as described in the Methods section and the indentation force, P, that we applied was determined using the following equation[1] :We generated a stretching force on a single neurite through indentation with a glass pipette into the soft PDMS surface. The indentation depth, h, was determined using a micromanipulator as described in the Methods section and the indentation force, P, that we applied was determined using the following equation[1] :
(0.07 MB TIF)

Figure S3 Lucifer yellow visualization of neurons during stretching (movie). A representative movie shows that a neurite-bearing DRG neuron (D2+F) was whole-cell patched and stretched by indenting the PDMS. The patched pipette was filled with internal solution containing 2 mg/ml Lucifer yellow. The duration of the indentation lasted for 0.52 seconds. The movie is composed of 13 frames.
(2.34 MB SWF)

Figure S4 Examining the effect of cytoskeleton modifiers on AP firing. (a) A current injection was introduced to evoke an AP in a DRG neuron perfused with ACSF. Nocodazole (1 µg/ml), which disrupts microtubules, did not block current injection-induced AP in DRG neurons (n = 6). (b) Cytochalasin-D (1 µg/ml), which disrupts actin filaments, did not block current injection-induced AP in DRG neurons (n = 6). (c) Latrunculin-A (1 µg/ml), which inhibits actin polymerization, did not block current injection-induced AP in DRG neurons (n = 6).
(0.06 MB TIF)

Figure S5 Direct indentation on soma evoked an AP response in all neurite-free neurons of D2 culture (n = 5). Indented depth from cell surface to the position that fired an AP response is indicated.
(0.05 MB TIF)

Table S1 Cell densities of DRG cultures for D5, D2, D2+F groups. Cell densities were estimated by counting the cells in 2–3 fields of a culture. Three independent DRG cultures were performed for each group. D5, 5-day culture with poly-L-lysine; D2, 2-day culture with poly-L-lysine; D2+F, 2-day culture with fibronectin.
(0.06 MB TIF)
