# Supplementary figures and images for: Understanding Sensory Nerve Mechanotransduction through Localized Elastomeric Matrix Control
Source: PLoS One. 2009 Jan 28;4(1):e4293. doi: 10.1371/journal.pone.0004293 (PMC2627935; doi:10.1371/journal.pone.0004293)

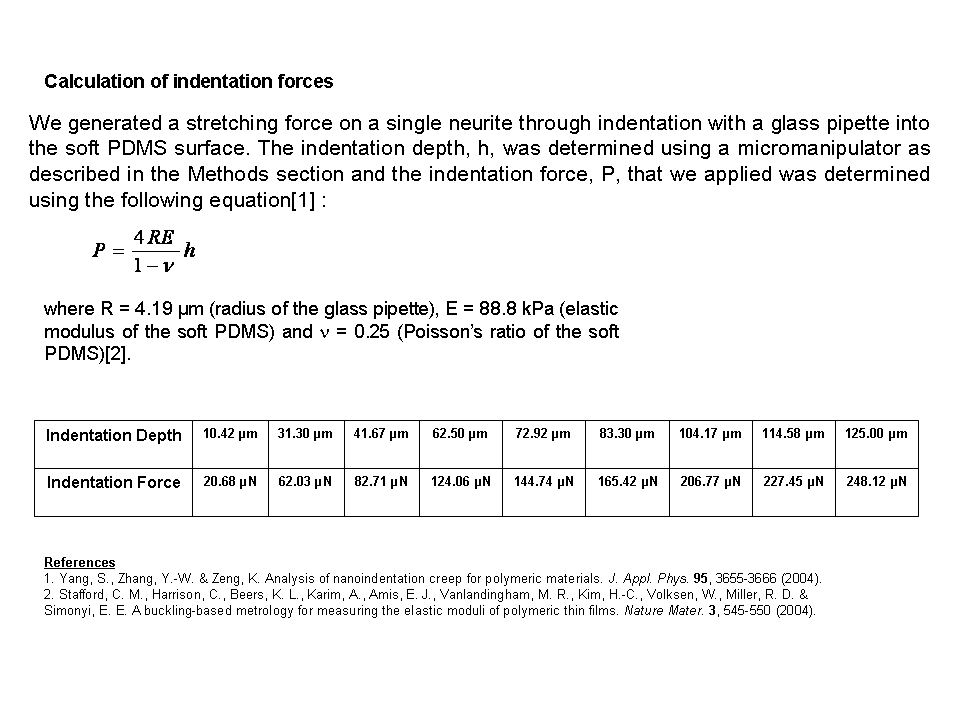

Supplement: Figure S2 — Calculation of indentation forces. We generated a stretching force on a single neurite through indentation with a glass pipette into the soft PDMS surface. The indentation depth, h, was determined using a micromanipulator as described in the Methods section and the indentation force, P, that we applied was determined using the following equation[1] :We generated a stretching force on a single neurite through indentation with a glass pipette into the soft PDMS surface. The indentation depth, h, was determined using a micromanipulator as described in the Methods section and the indentation force, P, that we applied was determined using the following equation[1] : (0.07 MB TIF) [file pone.0004293.s002.tif]

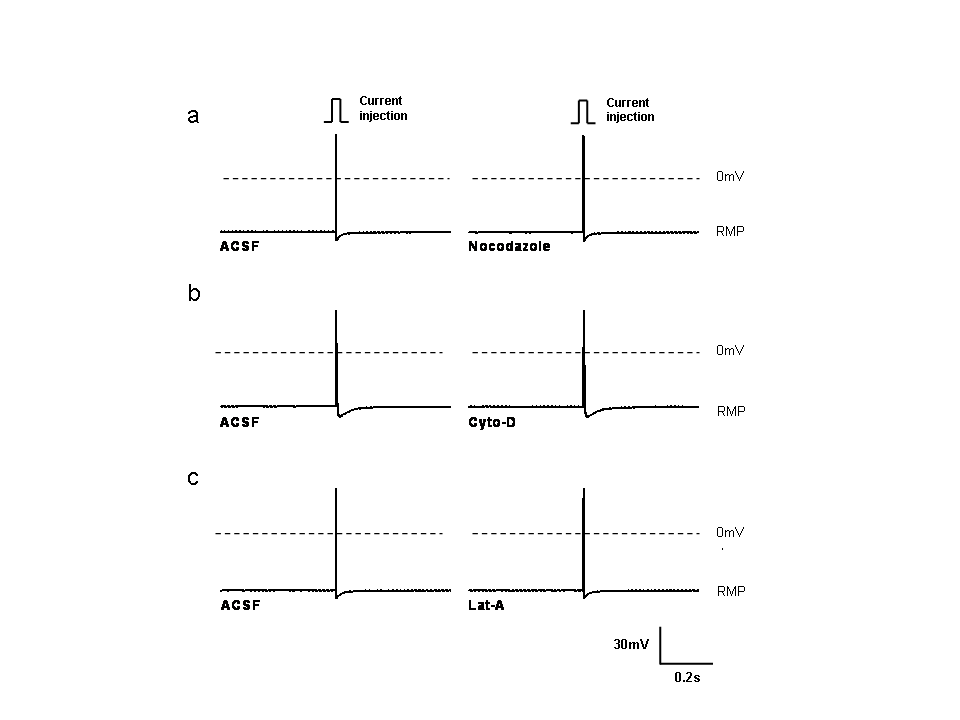

Supplement: Figure S4 — Examining the effect of cytoskeleton modifiers on AP firing. (a) A current injection was introduced to evoke an AP in a DRG neuron perfused with ACSF. Nocodazole (1 µg/ml), which disrupts microtubules, did not block current injection-induced AP in DRG neurons (n = 6). (b) Cytochalasin-D (1 µg/ml), which disrupts actin filaments, did not block current injection-induced AP in DRG neurons (n = 6). (c) Latrunculin-A (1 µg/ml), which inhibits actin polymerization, did not block current injection-induced AP in DRG neurons (n = 6). (0.06 MB TIF) [file pone.0004293.s004.tif]

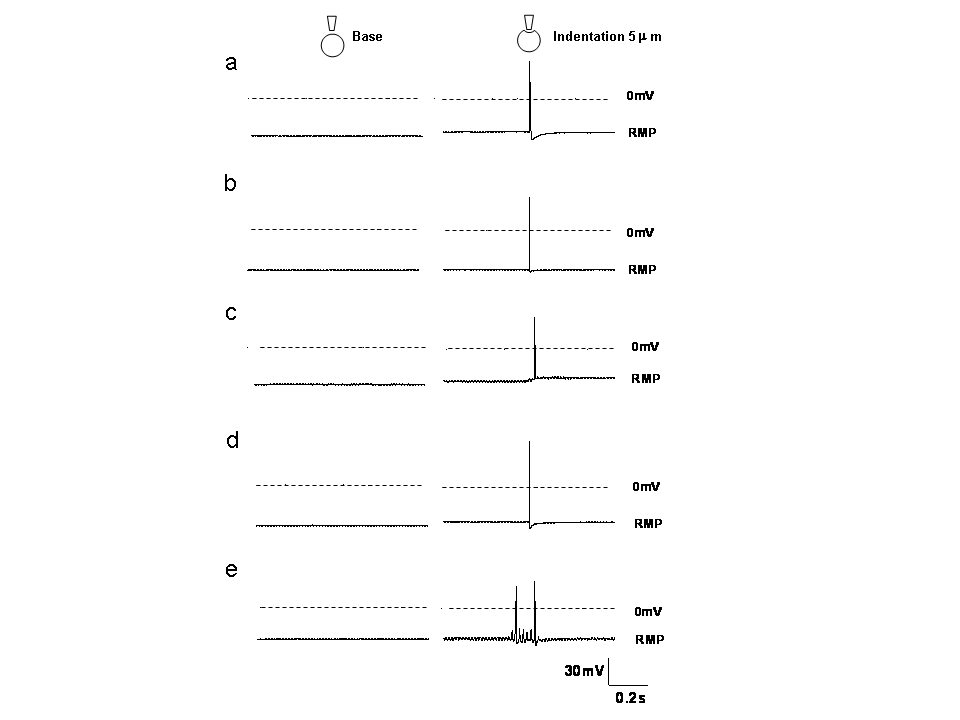

Supplement: Figure S5 — Direct indentation on soma evoked an AP response in all neurite-free neurons of D2 culture (n = 5). Indented depth from cell surface to the position that fired an AP response is indicated. (0.05 MB TIF) [file pone.0004293.s005.tif]

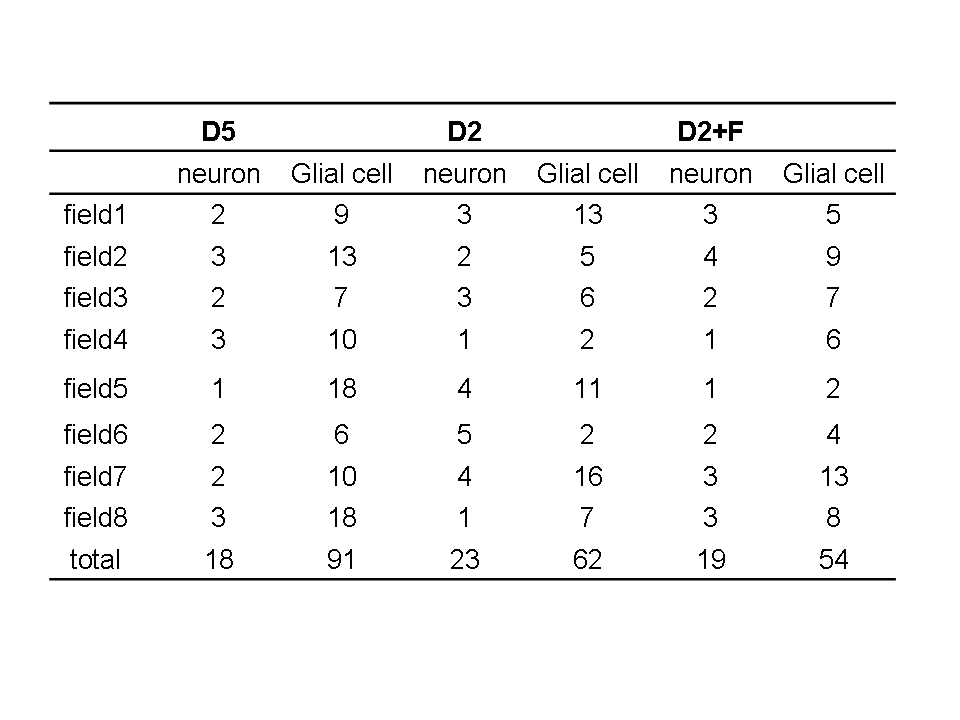

Supplement: Table S1 — Cell densities of DRG cultures for D5, D2, D2+F groups. Cell densities were estimated by counting the cells in 2–3 fields of a culture. Three independent DRG cultures were performed for each group. D5, 5-day culture with poly-L-lysine; D2, 2-day culture with poly-L-lysine; D2+F, 2-day culture with fibronectin. (0.06 MB TIF) [file pone.0004293.s006.tif]
